# Clinical significance of myeloperoxidase-anti-neutrophil cytoplasmic antibody in idiopathic interstitial pneumonias

**DOI:** 10.1371/journal.pone.0199659

**Published:** 2018-06-21

**Authors:** Hironao Hozumi, Yoshiyuki Oyama, Hideki Yasui, Yuzo Suzuki, Masato Kono, Masato Karayama, Kazuki Furuhashi, Noriyuki Enomoto, Tomoyuki Fujisawa, Naoki Inui, Yutaro Nakamura, Takafumi Suda

**Affiliations:** 1 Second Division, Department of Internal Medicine, Hamamatsu University School of Medicine, Hamamatsu, Shizuoka, Japan; 2 Department of Clinical Pharmacology and Therapeutics, Hamamatsu University School of Medicine, Hamamatsu, Shizuoka, Japan; Universitatsklinikum Freiburg, GERMANY

## Abstract

**Objective:**

Although a possible association among myeloperoxidase-anti-neutrophil cytoplasmic antibody (MPO-ANCA), microscopic polyangiitis (MPA), and idiopathic pulmonary fibrosis (IPF) has been suggested, the clinical significance of MPO-ANCA in idiopathic interstitial pneumonias (IIPs), including IPF and non-IPF, remains unclear. We aimed to investigate the frequency of MPO-ANCA positivity, as well as MPA incidence and risk factors for development in patients initially diagnosed with IIP.

**Methods:**

We retrospectively analysed 305 consecutive patients who were initially diagnosed as IIP and had MPO-ANCA results available.

**Results:**

Of the 305 patients, 26 (8.5%) were MPO-ANCA-positive. Baseline characteristics were similar between the MPO-ANCA-positive and -negative patients. The cumulative 5-year MPA incidence was 24.3% in the MPO-ANCA-positive patients and 0% in the -negative patients (*P* < 0.0001). MPO-ANCA was positive in 15 of 133 (11.3%) patients initially diagnosed with IPF and in 11 of 172 (6.3%) patients initially diagnosed with non-IPF (*P* = 0.56), with cumulative 5-year MPA incidence of 6.2% and 1.0%, respectively (*P* = 0.10). Multivariate analysis revealed that UIP pattern on HRCT (HR = 3.20, *P* < 0.01) and no treatment for IIP (HR = 3.52, *P* < 0.01) were independently associated with MPA development in MPO-ANCA-positive patients.

**Conclusion:**

MPO-ANCA positivity was uncommon, but was associated with subsequent MPA development in patients initially diagnosed with IIP, including both IPF and non-IPF cases. The study suggested that attention should be paid to MPA development in MPO-ANCA-positive IIP patients with UIP pattern on HRCT and those without treatment for IIP.

## Introduction

Idiopathic interstitial pneumonias (IIPs) comprise a spectrum of interstitial lung diseases (ILDs) of unknown etiology and are classified into several distinct disease entities, including idiopathic pulmonary fibrosis (IPF) [1–3]. The diagnosis of IIPs requires the exclusion of the secondary causes of ILD, particularly connective tissue disease (CTD). Therefore, the systemic evaluation of CTD-specific manifestations and autoantibodies is necessary to distinguish IIPs from CTD-ILD. However, this evaluation may detect patients with CTD-specific autoantibody but do not meet the established diagnostic criteria for a specific form of CTD. To solve this issue, the European Respiratory Society/American Thoracic Society task force has recently proposed the concept of interstitial pneumonia with autoimmune features (IPAF) [4]; however, the clinical significance of CTD-specific autoantibodies in patients with IIPs remains unclear.

Anti-neutrophil cytoplasmic antibodies (ANCAs), including myeloperoxidase-ANCA (MPO-ANCA), are a group of autoantibodies targeted against antigens in the cytoplasm of neutrophils. MPO-ANCA is detected predominantly in patients with ANCA-associated vasculitides, such as microscopic polyangiitis (MPA), granulomatosis with polyangiitis (GPA), and eosinophilic granulomatosis with polyangiitis (EGPA) [5–7]. MPA is a systemic, necrotizing vasculitis that primarily affects small vessels. Accumulating evidence suggested the possible association among MPO-ANCA, MPA and IPF. IPF patients who are positive for MPO-ANCA might include individuals in whom ILD precedes MPA [8–18]. However, in clinical practice, we occasionally encounter MPO-ANCA-positive patients with not only IPF but also with non-IPF types of IIPs. The clinical significance of MPO-ANCA in IIPs and the association between MPA and IIPs have not been fully elucidated. Of note, MPO-ANCA is not covered by the concept of IPAF because this antibody is associated with the vasculitides rather than with the CTD-ILD spectra of disorders [4]. To clarify these issues, we aimed to investigate the frequency of MPO-ANCA positivity, as well as the MPA incidence and risk factors for development in patients initially diagnosed with IIP, including IPF and non-IPF.

## Materials and methods

### Subjects

We retrospectively reviewed 321 consecutive patients who had been initially diagnosed with IIP between 2002 and 2016 at Hamamatsu University Hospital. Of the 321 patients, 16 were excluded because of the lack of available MPO-ANCA results during the study period. Consequently, 305 patients with the initial IIP diagnosis and who had available MPO-ANCA results were enrolled in this study. During this study period, these 305 patients were regularly followed up every 1–3 months. The patients’ medical records were assessed to obtain the clinical data, which included patient characteristics, laboratory data and pulmonary function at the time of diagnosis. The study was conducted in accordance with the Declaration of Helsinki. The institutional review board of Hamamatsu University School of Medicine approved this study (approval number 15–165) and waived patient approval or informed consent because the study involved a retrospective review of clinical records.

The diagnoses of IIPs, including IPF, idiopathic nonspecific interstitial pneumonia (NSIP), cryptogenic organizing pneumonia (COP), unclassifiable IIP and other IIPs, were based on clinical history, physical examination, and high-resolution computed tomography (HRCT) findings, with or without histologic examination, in accordance with international consensus criteria [1–3]. Chest HRCT images were reviewed by pulmonologists and chest radiologists, and the HRCT patterns were classified according to the 2011 IPF guidelines as usual interstitial pneumonia (UIP), possible UIP and inconsistent with UIP [2]. Lung specimens were reviewed by pathologists. The pathologic classifications of UIP, NSIP, COP, unclassifiable IIP and other IIPs were also based on the current guidelines [1–3]. At the time of initial diagnosis, all patients underwent systemic examination, including comprehensive autoantibody test and examination by rheumatologists, to exclude secondary causes of ILD and did not meet the established diagnostic criteria for any CTDs or systemic vasculitides.

ANCA-associated vasculitides, including MPA, were diagnosed according to the Chapel Hill consensus criteria [6, 7] and the European Medicines Agency algorithm [19] by a consensus among rheumatologists, pulmonologists and pathologists.

### Measurement of ANCA

Serum MPO-ANCA levels at the time of initial diagnosis and during follow-up were measured using an enzyme-linked immunosorbent assay kit with a cut-off level of 20 EU (2002–2012, NIPRO, Japan) or a chemiluminescent enzyme immunoassay kit with a cut-off level of 3.5 U/mL (2012–2017, MBL, Japan). In this study, MPO-ANCA levels were expressed as the ratio of MPO-ANCA titre to cut-off level because the cut-off levels varied according to the kits used during the study period. MPO-ANCA-positive patients were collectively defined as those in whom MPO-ANCA was positive at the time of initial evaluation and those in whom MPO-ANCA converted to positive during follow-up.

### Statistical analysis

All values were expressed as median (range) or number (%). The observation period was calculated from the date of initial IIP diagnosis until the last visit or time of death. The MPA-free observation period was calculated from the date of initial IIP diagnosis until the date of MPA development or until the last visit in patients who did not develop MPA. Fisher’s exact test was used for comparison of proportions among groups, whereas the Mann–Whitney U-test was used for comparing medians. Cox hazards analysis was used to identify variables associated with MPA development; all variables identified as significant in the univariate analysis were tested with multivariate analysis. Cumulative MPA incidence and survival were evaluated by the Kaplan–Meier method, and the curves were compared using the log-rank test. In all analyses, *P* < 0.05 was considered to indicate statistical significance. All data were analysed using commercially available software (JMP version 9.0.3a, SAS Institute Inc., NC, USA) and R software version 2.15.1 (The R Foundation for Statistical Computing, Austria).

## Results

### Frequency of MPO-ANCA positivity and MPA incidence in IIPs

**Fig 1:**
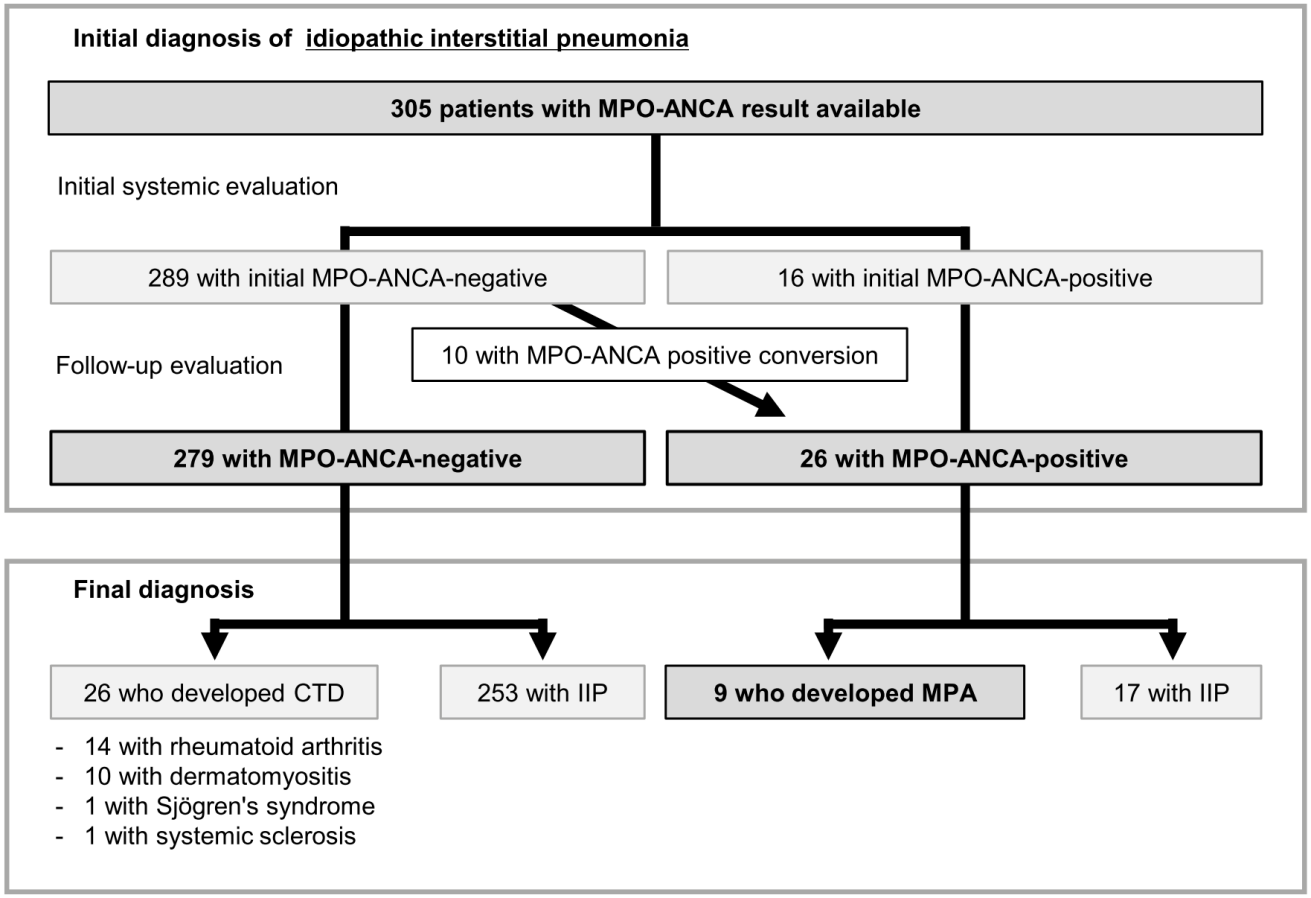
Flow chart of patient classification. CTD, connective tissue disease; IIPs, idiopathic interstitial pneumonias; MPA, microscopic polyangiitis; MPO-ANCA, myeloperoxidase-anti-neutrophil cytoplasmic antibody.

The flow chart for patient classification is presented in Fig 1. In all 305 patients initially diagnosed with IIP, the median observation period was 3.9 years [interquartile range (IQR), 2.0–6.5 years]. Of the 305 patients, 289 (94.8%) and 16 (5.2%) were MPO-ANCA-negative and -positive, respectively, at the time of initial systemic evaluation. During follow-up, 10 of the 289 MPO-ANCA-negative patients converted to MPO-ANCA-positive. In the 10 patients, the median time from the initial evaluation to MPO-ANCA positive-conversion was 2.3 years (IQR, 1.5–5.2 years). Consequently, 279 (91.5%) MPO-ANCA-negative and 26 (8.5%) MPO-ANCA-positive patients with the initial IIP diagnosis were analysed. Of the 279 MPO-ANCA-negative patients, none developed MPA over the study period. On the other hand, 9 of the 26 MPO-ANCA-positive patients subsequently developed MPA (Fig 1) after a median MPA-free observation period of 5.0 years (IQR, 2.3–7.0 years; range, 0.5–10.0 years). Of the 9 MPO-ANCA-positive patients who developed MPA, 7 were diagnosed based on the pathological confirmation and systemic manifestations (kidney biopsy in rapidly progressive glomerulonephritis, n = 4; skin biopsy in purpuric rash, n = 2; gastrointestinal mucosa biopsy in gastrointestinal bleeding, n = 1), and 2 were diagnosed on the basis of surrogate markers for renal vasculitis plus diffuse alveolar haemorrhage [19] (S1 Table). During the study period, no patient developed GPA or EGPA.

**Fig 2:**
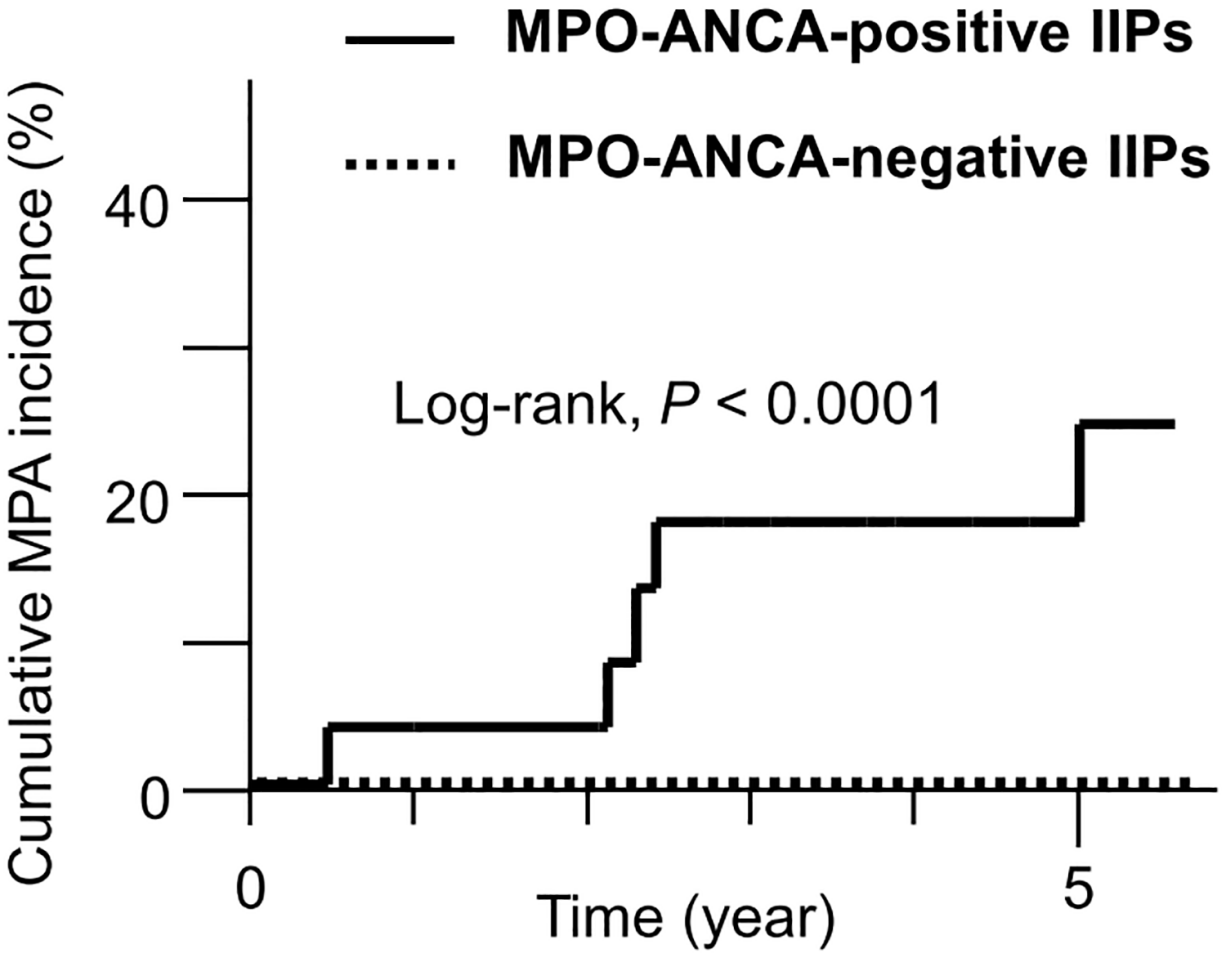
Cumulative MPA incidence in MPO-ANCA-positive and -negative IIP patients. The 5-year MPA incidence was 24.3% in the MPO-ANCA-positive IIP patients and 0% in the MPO-ANCA-negative IIP patients. *P* <0.0001 by log-rank test. IIPs, idiopathic interstitial pneumonias; MPA, microscopic polyangiitis; MPO-ANCA, myeloperoxidase-anti-neutrophil cytoplasmic antibody.

The cumulative MPA incidence rates in patients with the initial IIP diagnosis are shown in Fig 2. The MPO-ANCA-positive patients had a significantly higher 5-year cumulative MPA incidence compared with the MPO-ANCA-negative patients (24.3% vs. 0%, *P* < 0.0001).

### Subanalyses according to IIP classification

We divided the patients with the initial IIP diagnosis into those with IPF and those with non-IPF. The 5-year MPA incidence tended to be higher in the IPF group than in the non-IPF group (6.2% vs. 1.0%, *P* = 0.10).

**Fig 3:**
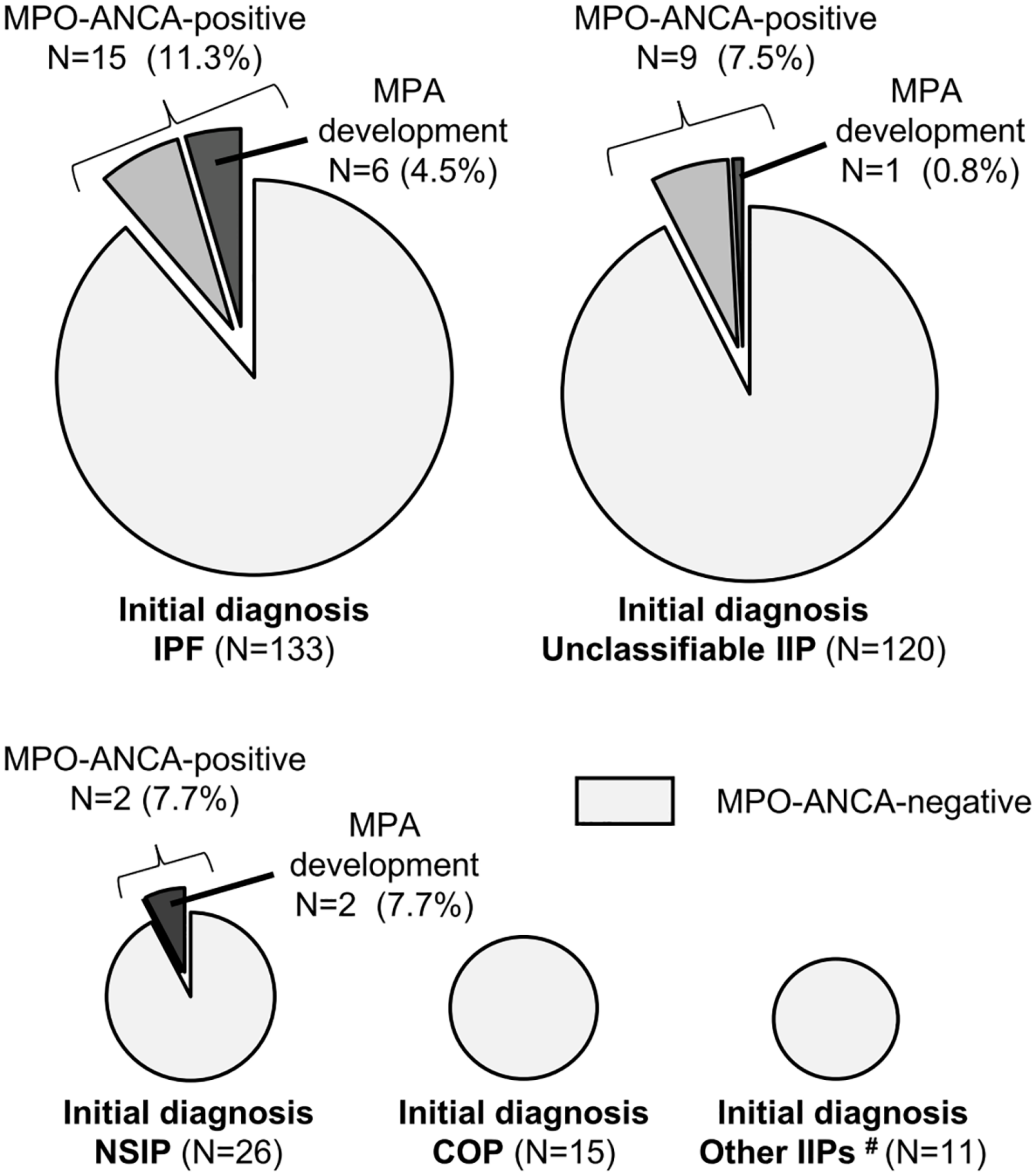
Frequencies of MPO-ANCA positivity and MPA development during the observation period in patients with the initial diagnoses of IPF, unclassifiable IIP, NSIP, COP and other IIPs (# AIP, n = 5; DIP/RB-ILD, n = 3; and PPFE, n = 3). AIP, acute interstitial pneumonia; COP, cryptogenic organizing pneumonia; DIP, desquamative interstitial pneumonia; IIPs, idiopathic interstitial pneumonias; IPF, idiopathic pulmonary fibrosis; MPA, microscopic polyangiitis; MPO-ANCA, myeloperoxidase-anti-neutrophil cytoplasmic antibody; NSIP, nonspecific interstitial pneumonia; PPFE, pleuroparenchymal fibroelastosis; RB-ILD, respiratory bronchiolitis-associated interstitial lung disease.

Fig 3 shows the frequency of MPO-ANCA positivity and MPA incidence according to the initial IIP diagnosis. MPO-ANCA positivity and the subsequent development of MPA were observed in patients with the initial diagnosrs of IPF, NSIP and unclassifiable IIP, but not in those with COP and other IIPs. Among the patients with initial diagnoses of IPF, NSIP and unclassifiable IIP, there were no significant differences in the frequency of MPO-ANCA positivity (*P* = 0.56) and MPA development (*P* = 0.10).

### Comparison between MPO-ANCA-positive and -negative patients

**Fig 4:**
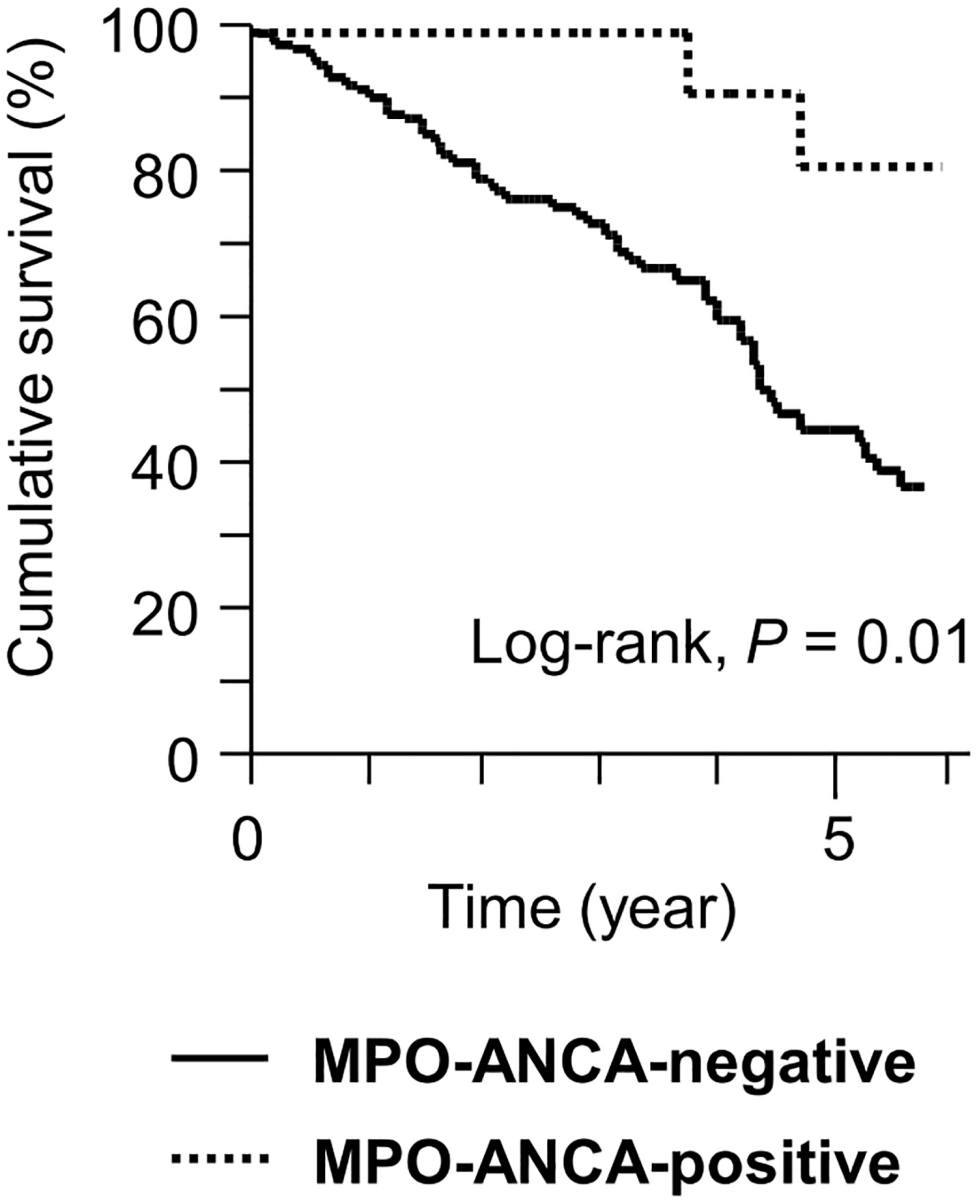
Cumulative survival rates of patients with the initial IPF diagnosis. The 5-year survival rate was 81.5% in the MPO-ANCA-positive patients, including patients who subsequently developed MPA, and 45.4% in the MPO-ANCA-negative patients. *P* = 0.01 by log-rank test. IPF, idiopathic pulmonary fibrosis; MPO-ANCA, myeloperoxidase-anti-neutrophil cytoplasmic antibody; MPA, microscopic polyangiitis.

In the IPF group, compared with MPO-ANCA-negative patients, MPO-ANCA-positive patients had significantly higher baseline serum KL-6 levels and frequency of MPA development (Table 1). There were no significant differences in the other baseline characteristics and clinical events. Moreover, the 5-year cumulative survival of the MPO-ANCA-positive patients, including those who developed MPA, was significantly higher than that of the MPO-ANCA-negative patients (81.5% vs. 45.4%, *P* = 0.01; Fig 4).

**Table 1:** Comparison of patients who were initially diagnosed as having IPF on the basis of MPO-ANCA results.

	MPO-ANCA-negative N = 118 (88.7%)	MPO-ANCA-positive N = 15 (11.3%)	*P*-value
**Baseline characteristics**			
Age, years	71 (64–76)	68 (61–77)	0.18
Male/Female	102 (86)/16 (14)	12 (80)/3 (20)	0.45
Current or former smoker	95 (81)	13 (87)	0.74
**Initial HRCT pattern**			
UIP/possible UIP	83 (70)/35 (30)	9 (60)/6 (40)	0.55
**Baseline laboratory data**			
CRP, mg/dL	0.2 (0.1–0.4)	0.5 (0.1–0.7)	0.07
KL-6, U/mL	882 (614–1221)	1597 (827–3030)	<0.01*
PaO_2_, Torr	77 (70–88)	76 (69–84)	0.54
**Baseline Pulmonary function**			
% FVC	73 (62–89)	81 (66–98)	0.11
FEV_1.0_ /FVC, %	84 (80–89)	82 (79–86)	0.45
**Surgical lung biopsy**	33 (28)	5 (33)	0.86
**Initial treatment regimen**			0.74
Immunosuppressive	26 (22)	3 (20)	
PSL monotherapy	13	2	
PSL + CPA	7	0	
PSL + CyA	6	1	
Anti-fibrotic	49 (42)	5 (33)	
Pirfenidone	43	4	
Nintedanib	6	1	
**Observation period, years**	3.4 (1.7–4.8)	5.9 (3.9–12.6)	<0.001*
**Clinical event**			
MPA development	0 (0)	6 (40)	<0.0001*
Death from all causes	60 (51)	8 (53)	0.99
Death from respiratory failure	51 (43)	8 (53)	0.58

Data are presented as n (%) or median (IQR).

*P < 0.05

CPA, cyclophosphamide; CRP, C-reactive protein; CyA, cyclosporine A; FEV_1.0_, forced expiratory volume in 1.0 s; FVC, forced vital capacity; HRCT, high-resolution computed tomography; IIP, idiopathic interstitial pneumonia; IPF, idiopathic pulmonary fibrosis; KL-6, Krebs von den Lungen-6; MPA, microscopic polyangiitis; PaO_2_, arterial oxygen pressure; PSL, prednisolone; UIP, usual interstitial pneumonia

In the non-IPF group, compared with the MPO-ANCA-negative patients, the MPO-ANCA-positive patients had significantly higher frequency of MPA development (Table 2) but had similar baseline characteristics, other clinical events and survival (S1 Fig).

**Table 2:** Comparison of patients who were initially diagnosed as having non-IPF on the basis of MPO-ANCA results.

	MPO-ANCA-negative N = 161 (93.6%)	MPO-ANCA-positive N = 11 (6.4%)	*P*-value
**Baseline characteristics**			
Age, years	69 (61–74)	72 (65–75)	0.35
Male/Female	96 (60)/65 (40)	8 (73)/3 (27)	0.53
Current or former smoker	93 (58)	8 (73)	0.53
**Initial IIP diagnosis**			0.67
NSIP	24 (15)	2 (18)	
COP	15 (9)	0 (0)	
Unclassifiable IIP	111 (69)	9 (82)	
Other IIPs^‡^	11 (7)	0 (0)	
**Baseline Laboratory data**			
CRP, mg/dL	0.2 (0.1–0.8)	0.5 (0.1–1.3)	0.62
KL-6, U/mL	784 (452–1378)	1215 (466–1769)	0.35
PaO_2_, Torr	78 (72–85)	69 (63–79)	0.05
**Baseline pulmonary function**			
% FVC	80 (67–93)	71 (61–97)	0.53
FEV_1.0_/FVC, %	83 (76–88)	80 (78–86)	0.65
**Surgical lung biopsy**	60 (38)	3 (27)	0.75
**Initial treatment**			0.75
Immunosuppressive	62 (39)	5 (45)	
PSL monotherapy	50	4	
PSL + CPA	6	1	
PSL + CyA	6	0	
Anti-fibrotic	6 (4)	0 (0)	
Pirfenidone	6	0	
Nintedanib	0	0	
**Observation period, years**	4.1 (1.9–8.0)	5.6 (2.1–8.2)	0.51
**Clinical event**			
MPA development	0 (0)	3 (27)	<0.001*
Death from all causes	45 (28)	5 (45)	0.30
Death from respiratory failure	30 (22)	4 (36)	0.25

Data are presented as n (%) or median (IQR).

*P < 0.05

CPA, cyclophosphamide; CRP, C-reactive protein; COP, cryptogenic organizing pneumonia; CyA, cyclosporine A; FEV_1.0_, forced expiratory volume in 1.0 s; FVC, forced vital capacity; HRCT, high-resolution computed tomography; IIP, idiopathic interstitial pneumonia; IPF, idiopathic pulmonary fibrosis; KL-6, Krebs von den Lungen-6; MPA, microscopic polyangiitis; NSIP, nonspecific interstitial pneumonia; PaO_2_, arterial oxygen pressure; PSL, prednisolone; UIP, usual interstitial pneumonia

^‡^Acute interstitial pneumonia (n = 5), desquamative interstitial pneumonia/respiratory bronchiolitis-associated interstitial lung disease (n = 3), pleuroparenchymal fibroelastosis (n = 3)

### Risk factor for MPA development and mortality in MPO-ANCA-positive IIP patients

To analyse the risk factors for MPA development and mortality in MPO-ANCA-positive patients initially diagnosed with IIP, we compared the characteristics between those who developed MPA (MPA group) and those who did not develop MPA (non-MPA group) and performed cox hazards analysis.

As shown in Table 3, compared with the non-MPA group, the MPA group had significantly higher frequency of UIP pattern on HRCT (67% vs. 18%, *P* = 0.03) at the time of initial diagnosis and significantly differed in the initial treatment for IIP (*P* <0.01). Kaplan–Meier survival curves demonstrated that the 5-year cumulative survival rate was 87.5% in the MPA group and 74.1% in the non-MPA group (*P* = 0.52 by log-rank test).

**Table 3:** Comparison of MPO-ANCA-positive patients according to the development of MPA.

	MPA N = 9	Non-MPA N = 17	P-value
**Baseline characteristics**			
Age, years	68 (61–76)	68 (64–77)	0.55
Male / Female	6 (67) / 3 (33)	14 (82) / 3 (18)	0.63
Current or former smoker	8 (89)	13 (76)	0.63
Initial diagnosis, IPF/non-IPF	6 (67)/3 (33)	9 (53)/8 (47)	0.68
**Baseline laboratory data**			
CRP, mg/dL	0.6 (0.3–0.7)	0.4 (0.1–1.0)	0.82
KL-6, U/mL	1465 (470–3243)	1320 (780–1772)	0.82
PaO_2_, Torr	76 (66–78)	74 (64–83)	0.83
MPO-ANCA			
Positive at initial IIP diagnosis/positive conversion	4 (44) / 5 (56)	12 (71) / 5 (29)	0.23
Titre/cut-off at initial IIP diagnosis, ratio	3.4 (2.0–6.9)	1.5 (1.2–2.5)	0.07
**Baseline pulmonary function**			
% FVC	75 (65–101)	76 (65–95)	0.69
FEV_1.0_/FVC, %	81 (78–87)	82 (78–86)	0.73
**Initial HRCT pattern**			<0.01*
UIP	6 (67)	3 (18)	0.03*
Possible UIP	0	10 (59)	
Inconsistent with UIP	3 (33)	4 (23)	
**Surgical lung biopsy**	4 (44)	4 (24)	0.38
**Initial treatment**			0.01*
Immunosuppressive	0 (0)	8 (47)	
PSL monotherapy	0	6	
PSL + CPA	0	1	
PSL + CyA	0	1	
Anti-fibrotic	1 (11)	4 (24)	
Pirfenidone	0	4	
Nintedanib	1	0	
None	8 (89)	5 (29)	
**MPA-free observation period, years**	5.0 (2.3–7.0)	5.6 (3.0–9.9)	0.48
**Observation period, years**	6.1 (4.0–9.2)	5.6 (3.0–9.9)	0.55
**Death from all causes**	6 (67)	7 (41)	0.41
**Death from respiratory failure**	6 (67)	6 (35)	0.21

Data are presented as n (%) or median (IQR).

*P < 0.05

CPA, cyclophosphamide; CRP, C-reactive protein; CyA, cyclosporine A; %DLCO, predicted diffusing capacity of the lung for carbon monoxide; FEV_1.0_, forced expiratory volume in 1.0 s; FVC, forced vital capacity; IIP, idiopathic interstitial pneumonia; IPF, idiopathic pulmonary fibrosis; KL-6, Krebs von den Lungen-6; MPA, microscopic polyangiitis; PaO_2,_ arterial oxygen pressure; PSL, prednisolone; UIP, usual interstitial pneumonia

The results of multivariate cox hazard analysis for MPA development revealed that in MPO-ANCA-positive patients, UIP pattern on HRCT at the time of initial IIP diagnosis and no treatment for IIP were independently associated with a higher risk for subsequent MPA development (Table 4).

**Table 4:** Cox hazard analysis for MPA development in MPO-ANCA-positive patients.

	HR	95% CI	*P*-value
**Univariate**			
Male (vs. female)	0.47	0.20–1.11	0.08
Age, years	1.04	0.97–1.13	0.26
Smoking, yes	0.70	0.26–3.11	0.55
Initial IPF diagnosis, yes	1.14	0.58–2.48	0.71
MPO-ANCA titre/cut-off ratio at the initial IIP diagnosis	1.02	0.98–1.05	0.27
MPO-ANCA positive conversion	2.69	0.70–11.1	0.15
PaO_2_, Torr	0.93	0.86–1.01	0.08
% FVC, %	0.99	0.95–1.03	0.78
FEV_1.0_/FVC, %	1.001	0.90–1.13	0.98
CRP, mg/dL	0.81	0.23–1.32	0.55
KL-6, U/mL	1.00	0.999–1.001	0.77
UIP pattern on HRCT at initial diagnosis, yes	2.64	1.25–6.94	<0.01*
No treatment for the initial IIP diagnosis^‡^	2.98	1.27–12.8	<0.01*
**Multivariate**			
UIP pattern on HRCT at initial diagnosis, yes	3.20	1.41–9.57	<0.01*
No treatment for the initial IIP diagnosis^‡^	3.52	1.42–15.9	<0.01*

*P < 0.05

CRP, C-reactive protein; FEV_1.0_, forced expiratory volume in 1.0 second; FVC, forced vital capacity; HRCT, high-resolution computed tomography; IIP, idiopathic interstitial pneumonia; IPF, idiopathic pulmonary fibrosis; KL-6, Krebs von den Lungen-6; PaO_2,_ arterial oxygen pressure;

^‡^Before MPA development in the MPA group

Although the univariate Cox hazard analysis for mortality revealed that older age (HR 1.09, *P* = 0.01), lower % FVC (HR 0.94, *P* < 0.01) and higher FEV_1.0_/FVC (HR 1.12, *P* = 0.03) were associated with mortality, only older age was independently associated with a poorer prognosis in multivariate analysis (HR 1.08, *P* = 0.04) (S2 Table).

## Discussion

In this study, we found that 8.5% of patients with IIP were positive for serum MPO-ANCA at the time of initial diagnosis or seroconverted to positive during follow-up; this MPO-ANCA positivity was associated with subsequent development of MPA. In the MPO-ANCA-positive IIP patients, the 5-year cumulative MPA incidence was 24.3%. The potential to develop MPA in MPO-ANCA-positive patients was observed not only in those with the initial IPF diagnosis but also in those with non-IPF, and that this risk was independently associated with UIP pattern on HRCT and the absence of treatment for IIP. In the IPF group, the survival of the MPO-ANCA-positive patients, including those who developed MPA, was significantly higher than that of the MPO-ANCA-negative patients. To the best of our knowledge, this is the first study to report the association among MPO-ANCA, MPA, and IIPs, including both IPF and non-IPF cases.

Previous reports showed that 7% to 15% of patients initially diagnosed with IPF were either MPO-ANCA-positive upon initial diagnosis or seroconverted during follow-up, and that approximately 25% of the MPO-ANCA-positive patients developed MPA [13, 14, 18]. Consistent with these reports, the current study demonstrated that 11.3% of patients with the initial IPF diagnosis were MPO-ANCA-positive and that 40% of these MPO-ANCA-positive patients subsequently developed MPA. However, there has been no study that investigated the frequency of MPO-ANCA positivity and MPA incidence in patients with non-IPF. In the current study, 6.4% of patients with the initial diagnosis of non-IPF, including NSIP and unclassifiable IIP, were MPO-ANCA-positive; 27% of these MPO-ANCA-positive patients developed MPA. Although we analyzed the risk factors for the seroconversion of MPO-ANCA, no significant factor was identified (data not shown). Collectively, the current study demonstrated that in MPO-ANCA-positive patients, those with the initial diagnosis of non-IPF, particularly NSIP and unclassifiable IIP, had a similar potential to develop MPA as those with IPF. These results suggested that ILDs preceding MPA may mimic several subsets of IIPs at the time of initial presentation.

The current study demonstrated that in patients initially diagnosed with IIP, serum MPO-ANCA positivity was associated with subsequent MPA development. Further analyses showed that in patients initially diagnosed with IIP, including both IPF and non-IPF, those with positive MPO-ANCA were similar to those with negative MPO-ANCA in terms of clinical features, except MPA incidence. Notably, IPF patients who were MPO-ANCA-positive had higher 5-year survival rates than those who were MPO-ANCA-negative. In this cohort, MPO-ANCA-positive IPF patients tended to be younger and presented with higher FVC at the baseline compared with MPO-ANCA-negative IPF patients. These factors may explain the survival difference between the two groups. However, another study showed a similar trend in survival [18]. Previous studies demonstrated that the pathologic features of the lung were different between MPO-ANCA-positive and -negative patients [15, 18]. Considering the differences in MPA incidence, survival and pathologic features, there should be a distinction between MPO-ANCA-positive and MPO-ANCA-negative patients with IIP, especially those with IPF. Interestingly, MPO-ANCA-positive IIP might be a distinct ILD or MPA variant. Although MPO-ANCA is not included in the concept of IPAF [4], these data may support the importance of evaluating autoimmune features in patients with IIP.

To date, there has been no study that identified the risk factors for MPA development in MPO-ANCA-positive patients initially diagnosed with IIP. In the current study, the independent risk factor for MPA development in these patients was not the diagnosis of IPF *per se*, but a UIP pattern on HRCT. The diagnosis of IPF requires either the presence of the typical UIP pattern on HRCT or specific combinations of lung biopsy findings and HRCT patterns, such as possible UIP [2]. Reportedly, the most frequent HRCT pattern in MPA–ILD patients was UIP [8, 9, 12, 20]. Moreover, UIP appeared to be the predominant HRCT pattern in MPO-ANCA-positive IPF, especially in cases of ILD preceding MPA [8, 11, 13]. The observations suggested a closer association of MPA with UIP pattern on HRCT than with UIP proven by lung biopsy and possible UIP on HRCT.

Several studies suggested that immunosuppressive therapy can reduce the risk of MPA development in MPO-ANCA-positive patients initially diagnosed with IPF [13, 14, 18]. In other words, patients who are not administered immunosuppressive therapy might have a relatively high risk for MPA development. In the current study, the absence of immunosuppressive or anti-fibrotic treatment for IIP was an independent risk factor for MPA development in MPO-ANCA-positive patients with the initial IIP diagnosis. To date, there has been no established treatment protocol for IIPs with autoimmune features. A prospective study is warranted to verify the clinical effectiveness of immunosuppressive or anti-fibrotic treatment for MPO-ANCA-positive IIP patients.

This study had several limitations. First, the retrospective design subjected this study to several possible biases. For instance, because our institution is a regional ILD referral centre, referral or selection bias may have existed. Second, although MPO-ANCA was routinely and repeatedly examined, at least more than twice, but it was not regularly measured during the observation period (at approximately every 3–12 months depending on attending physicians). Therefore, it is possible that patients with incidental or occult MPO-ANCA positivity may have been excluded from the MPO-ANCA-positive group of patients. However, the prevalence of MPO-ANCA positivity is considered to be rare in IIPs and during the current study period, none of the MPO-ANCA-negative patients developed MPA. Therefore, it may not have significantly affected our results. Third, because of the small sample size of the MPO-ANCA-positive patients, the results of the multivariate analysis of risk factors for MPA development should be carefully interpreted. Finally, the different treatment regimens administered during the study period might have affected the survival rates of the groups analysed.

In conclusion, although MPO-ANCA positivity was uncommon, it was associated with the subsequent development of MPA in patients initially diagnosed with IIP, including both non-IPF and IPF cases. Particular attention should be paid to MPA development in MPO-ANCA-positive IIP patients with UIP pattern on HRCT and those who do not receive IIP treatment. These results may warrant further distinction between MPO-ANCA-positive and -negative IIP patients at the time of initial diagnosis and during the follow-up and will provide valuable information to rheumatologists and pulmonologists in the clinical practice. Nonetheless, prospective and larger studies are needed to validate our results.

## Supporting information

S1 Fig Cumulative survival in patients with the initial diagnosis of non-IPF. The 5-year survival rate was 70% in the MPO-ANCA-positive patients, including patients who subsequently developed MPA, and 74.4% in the MPO-ANCA-negative patients. *P* = 0.29 by log-rank test. IPF, idiopathic pulmonary fibrosis; MPO-ANCA, myeloperoxidase-anti-neutrophil cytoplasmic antibody; MPA, microscopic polyangiitis.
(TIF)

S1 Table Organs involved in patients who developed MPA, except for lung. (DOCX)

S2 Table Cox hazard analysis for mortality in MPO-ANCA-positive patients. (DOCX)
