# Supplementary material for: Clinical significance of myeloperoxidase-anti-neutrophil cytoplasmic antibody in idiopathic interstitial pneumonias
Source: PLoS One. 2018 Jun 21;13(6):e0199659. doi: 10.1371/journal.pone.0199659 (PMC6013167; doi:10.1371/journal.pone.0199659)
Supplement: S1 Table — (DOCX) [file pone.0199659.s002.docx]

**S1 Table. Organs involved in patients who developed MPA, except for the lung**

| **Involved organ** |  |
| --- | --- |
| Kidney ^‡^ | 6 (67) |
| Skin ^§^ | 2 (22) |
| Gastrointestinal tract ^§^ | 1 (11) |

‡ Pathological confirmation of vasculitis (n = 4) and surrogate marker-positive for renal vasculitis (n = 2)

§ Pathological confirmation of vasculitis
