# Supplementary material for: Clinical significance of myeloperoxidase-anti-neutrophil cytoplasmic antibody in idiopathic interstitial pneumonias
Source: PLoS One. 2018 Jun 21;13(6):e0199659. doi: 10.1371/journal.pone.0199659 (PMC6013167; doi:10.1371/journal.pone.0199659)
Supplement: S2 Table — (DOCX) [file pone.0199659.s003.docx]

**S2 Table. Cox hazard analysis for mortality in MPO-ANCA-positive patients.**

|  | **HR** | **95% CI** | ***P*-value** |
| --- | --- | --- | --- |
| **Univariate** |  |  |  |
| Male (vs. female) | 0.44 | 0.12–2.11 | 0.28 |
| Age, year | 1.09 | 1.02–1.18 | 0.01* |
| Smoking, yes | 0.20 | 0.05–1.01 | 0.06 |
| Initial diagnosis of IPF, yes | 0.92 | 0.30–3.09 | 0.89 |
| MPO-ANCA titre / cut-off ratio at the initial IIP diagnosis | 0.91 | 0.70–1.01 | 0.13 |
| MPO-ANCA positive conversion | 1.07 | 0.35–3.59 | 0.91 |
| MPA development | 0.70 | 0.23-2.20 | 0.53 |
| PaO_2_, Torr | 0.95 | 0.88–1.01 | 0.12 |
| % FVC, % | 0.94 | 0.89–0.98 | <0.01* |
| FEV_1.0_/FVC, % | 1.12 | 1.01–1.27 | 0.03* |
| CRP, mg/dL | 0.91 | 0.41–1.35 | 0.75 |
| KL-6, U/mL | 1.00 | 0.99–1.0003 | 0.57 |
| UIP pattern on HRCT at initial diagnosis, yes | 1.29 | 0.38–4.00 | 0.67 |
| No treatment for initial diagnosis of IIP ^‡^ | 1.23 | 0.38–4.28 | 0.73 |
| **Multivariate** |  |  |  |
| Age, year | 1.08 | 1.004–1.17 | 0.04* |
| % FVC, % | 0.96 | 0.90–1.01 | 0.14 |
| FEV_1.0_/FVC, % | 1.05 | 0.92–1.23 | 0.95 |

**P* < 0.05

CRP, C-reactive protein; FEV_1.0_, forced expiratory volume in 1.0 second; FVC, forced vital capacity; HRCT, high-resolution computed tomography; IIP, idiopathic interstitial pneumonia; IPF, idiopathic pulmonary fibrosis; KL-6, Krebs von den Lungen-6; PaO_2,_ arterial oxygen pressure;

‡ Before MPA development in the MPA group
